# Comparison of non-invasive to invasive oxygenation ratios for diagnosing acute respiratory distress syndrome following coronary artery bypass graft surgery: a prospective derivation-validation cohort study

**DOI:** 10.1186/s13019-018-0804-8

**Published:** 2018-11-27

**Authors:** Farshid R. Bashar, Amir Vahedian-Azimi, Behrooz Farzanegan, Reza Goharani, Seyedpouzhia Shojaei, Sevak Hatamian, Seyed M. M. Mosavinasab, Masoum Khoshfetrat, Mohammad A. K. Khatir, Anna Tomdio, Andrew C. Miller, for the MORZAK Collaborative

**Affiliations:** 1 Anesthesia and Critical Care Department, Hamadan University of Medical Sciences, Hamadan, Iran; 2 Trauma Research Center, Nursing Faculty, Baqiyatallah University of Medical Sciences, Tehran, Iran; 3 Tracheal Diseases Research Center, Anesthesia and Critical Care Department, Masih Daneshvari Hospital, Shahid Beheshti University of Medical Sciences, Tehran, Iran; 4 Anesthesiology Research Center, Anesthesia and Critical Care Department, Loghman Hakim Hospital, Shahid Beheshti University of Medical Sciences, Tehran, Iran; 5 Anesthesia and Critical Care Department, Alborz University of Medical Sciences, Karaj, Iran; 6 Anesthesiology Research Center, Anesthesia and Critical Care Department, Modares Hospital, Shahid Beheshti University of Medical Sciences, Tehran, Iran; 7 Anesthesiology Research Center, Anesthesia and Critical Care Department, Khatam-o-anbia Hospital, Zahedan University of Medical Sciences, Zahedan, Iran; 8 Anesthesiology Research Center, Anesthesia and Critical Care Department, Taleghani Hospital, Shahid Beheshti University of Medical Sciences, Tehran, Iran; 9 Department of Internal Medicine, Vident Medical Center, East Carolina University Brody School of Medicine, Greenville, NC USA; 10 Department of Emergency Medicine, Vident Medical Center, East Carolina University Brody School of Medicine, 600 Moye Blvd, Greenville, NC 27834 USA

**Keywords:** Coronary artery bypass graft surgery, Acute respiratory distress syndrome, Oxygenation indices, Ratio of arterial oxygen partial pressure to fractional inspired oxygen, Ratio of peripheral capillary oxygen saturation to fractional inspired oxygen, Ratio of partial pressure of alveolar oxygen to fractional inspired oxygen

## Abstract

**Objective:**

To determine if non-invasive oxygenation indices, namely peripheral capillary oxygen saturation (S_p_O_2_)/fraction of inspired oxygen (F_i_O_2_) and partial pressure of alveolar oxygen (P_A_O_2_)/F_i_O_2_ may be used as effective surrogates for the partial pressure of arterial oxygen (P_a_O_2_)/F_i_O_2_. Also, to determine the S_p_O_2_/F_i_O_2_ and P_A_O_2_/F_i_O_2_ values that correspond to P_a_O_2_/F_i_O_2_ thresholds for identifying acute respiratory distress syndrome (ARDS) in patients following coronary artery bypass graft (CABG) surgery.

**Methods:**

A prospective derivation-validation cohort study in the Open-Heart ICU of an academic teaching hospital. Recorded variables included patient demographics, ventilator settings, chest radiograph results, and S_P_O_2_, P_a_O_2_, P_A_O_2,_ S_a_O_2_, and F_i_O_2_. Linear regression modeling was used to quantify the relationship between indices. Receiver operating characteristic (ROC) curves were used to determine the sensitivity and specificity of the threshold values.

**Results:**

One-hundred seventy-five patients were enrolled in the derivation cohort, and 358 in the validation cohort. The S_P_O_2_/F_i_O_2_ and P_A_O_2_/F_i_O_2_ ratios could be predicted well from P_a_O_2_/F_i_O_2_, described by the linear regression models S_P_O_2_/F_i_O_2_ = 71.149 + 0.8PF and P_A_O_2_/F_i_O_2_ = 38.098 + 2.312PF, respectively. According to the linear regression equation, a P_a_O_2_/F_i_O_2_ ratio of 300 equaled an S_P_O_2_/F_i_O_2_ ratio of 311 (R^2^ 0.857, F 1035.742, < 0.0001) and a P_A_O_2_/F_i_O_2_ ratio of 732 (R^2^ 0.576, F 234.887, < 0.0001). The S_P_O_2_/F_i_O_2_ threshold of 311 had 90% sensitivity, 80% specificity, LR+ 4.50, LR- 0.13, PPV 98, and NPV 42.1 for the diagnosis of mild ARDS. The P_A_O_2_/F_i_O_2_ threshold of 732 had 86% sensitivity, 90% specificity, LR+ 8.45, LR- 0.16, PPV 98.9, and NPV 36 for the diagnosis of mild ARDS. S_P_O_2_/F_i_O_2_ had excellent discrimination ability for mild ARDS (AUC ± SE = 0.92 ± 0.017; 95% CI 0.889 to 0.947) as did P_A_O_2_/F_i_O_2_ (AUC ± SE = 0.915 ± 0.018; 95% CI 0.881 to0.942).

**Conclusions:**

P_a_O_2_ and S_a_O_2_ correlated in the diagnosis of ARDS, with a P_a_O_2_/F_i_O_2_ of 300 correlating to an S_P_O_2_/ F_i_O_2_ of 311 (Sensitivity 90%, Specificity 80%). The S_P_O_2_/ F_i_O_2_ ratio may allow for early real-time rapid identification of ARDS, while decreasing the cost, phlebotomy, blood loss, pain, skin breaks, and vascular punctures associated with serial arterial blood gas measurements.

## Background

Circulatory and cardiovascular diseases (CVD) remain the leading cause of death globally [1]. In 2013, 17 million (32%) of NCD deaths were attributable to CVD, a number expected to rise to 11.1 million by 2020 [3], and > 23.6 million by 2030 [3–5]. Coronary artery disease (CAD), the most common type of CVD, is the leading cause of death, morbidity and decline in quality-of-life (QoL) globally, and is predicted to remain so for the next 20 years [2, 3].

Coronary artery bypass graft (CABG) surgery is a common revascularization technique used to re-establish or improve flow to under-perfused regions of the heart. Predicting outcomes in postoperative cardiac surgery patients has proven to be an extremely difficult task. The severity-of-illness scoring systems that are commonly used in medical ICUs have not been very useful in predicting death in these patients, as high scores are often not associated with poor outcome [6–8]. This may in part be due to our ability to normalize physiology by means of pharmacologic and/or mechanical support. The reported incidence of acute respiratory distress syndrome (ARDS) in patients undergoing open heart surgery with cardiopulmonary bypass (CPB) is 0.4–2.5%, with an associated mortality rate up to 68.4% [9–12]. The current ARDS definition (Berlin Criteria) requires arterial blood gas (ABG) measurement to monitor the partial pressure of O_2_ in arterial blood (P_a_O_2_) and includes a minimum PEEP value to account for the effect of mechanical ventilator settings on P_a_O_2_/F_i_O_2_ [13]. However, the Berlin definition is only slightly better than its predecessor for ARDS prognostication (receiver operating characteristic [ROC] area under the curve [AUC], 0.577 vs 0.536). This may be partially related to the dependence of both definitions on P_a_O_2_/F_i_O_2_ as the primary measure of ARDS severity. Multiple studies have shown that P_a_O_2_/F_i_O_2_ is not an independent mortality predictor in ARDS [14–16]. The P_a_O_2_/F_i_O_2_ does not reflect other aspects of lung injury severity such as mechanical ventilation settings, changes in lung compliance, and pulmonary shunt [17]. Additionally, concerns about anemia, excessive phlebotomy, and a movement to minimally invasive approaches have led to fewer ABG measurements in critically ill patients [18]. We investigated the performance of the peripheral capillary oxygen saturation (S_p_O_2_)/F_i_O_2_ and partial pressure of alveolar O_2_ (P_A_O_2_)/F_i_O_2_ oxygenation indices to P_a_O_2_/F_i_O_2_ (P/F) in identifying post-operative ARDS in CABG patients. Additionally, we seek to determine the threshold values for S_p_O_2_/F_i_O_2_ (S/F) and P_A_O_2_/F_i_O_2_ (P_A_/F) that correlate with P/F ratios consistent with ARDS (mild 201–300; moderate 101–200; severe ≤100 mmHg).

## Methods

### Study design and setting

We conducted a prospective derivation-validation cohort study in the Open-Heart ICU of an academic teaching hospital from October 1, 2011 to April 31, 2012. All patients undergoing CABG surgery during this period were screened for enrollment. The study protocol was approved by the local institutional investigative review board. All study parts were reviewed according to the Strengthening the Reporting of Observational Studies in Epidemiology (STROBE) Statement: guidelines for reporting observational studies [19]**.**

Patients were eligible for study participation if: (1) age ≥ 18 years, (2) full code-status, (3) first CABG surgery, (4) non-emergency CABG Surgery, (5) left ventricular ejection fraction ≥30%, and (6) informed consent provided by the patient, legal guardian, or healthcare surrogate.

Patients were excluded for: (1) post-operative hemorrhage > 300 ml in the first hour post-op with need for Surgical intervention; (2) unscheduled return to surgery; (3) pacemaker dependence; (4) history of prior CABG; (5) emergency CABG; (6) left ventricular ejection fraction (LVEF) < 30%; (7) psychiatric comorbidity; or (8) excessive sedation requirements. Sedation levels were determined in accordance with published recommendations [20–22].

Recorded variables included patient demographics, ventilator settings, chest radiograph results, and S_P_O_2_, P_a_O_2_, F_i_O_2_ and S_a_O_2_. These latter were used to calculate S/F, P_A_/F and P/F ratios.

### Data sources

#### Derivation group

Corresponding measurements of S_P_O_2_ and P_a_O_2_ from patients (*n* = 175) undergoing CABG surgery were utilized to establish the relationship between S/F, P_A_/F and P/F ratios. All mechanical ventilation settings were performed by three intensivists, and settings were consistent with routine standard-of-care including low V_T_ ventilation strategies. All patients were ventilated using Dräger Evita® XL or Evita® 4 ventilators (Dräeger Medical, Inc., Lubeck, Germany).

S_P_O_2_, P_a_O_2_, and F_i_O_2_ were measured once per patient upon study enrollment, and S_P_O_2_ was recorded at the time of ABG sampling. In rare cases when this was not possible, the S_a_O_2_ measurement closest temporally to the S_P_O_2_ value was utilized. To optimize S_P_O_2_ measurements, investigators utilized optimal patient and sensor position, ensured sensor cleanliness, and used only measurements with satisfactory waveforms. No position changes or endobronchial suctioning was performed within 10 min of measurement, and no invasive procedures or ventilator changes were performed within 30 min of measurement [12]. S_P_O_2_ was observed for a minimum of 1 min before the value was recorded. Like other studies, measurements with S_P_O_2_ > 97% were excluded from analysis because the oxyhemoglobin dissociation curve is flat above these levels [18].

#### Validation group

Corresponding measurements of S_P_O_2_ and P_a_O_2_ from patients undergoing CABG surgery (*n* = 358) were used to establish the relationship between S/F, P_A_/F and P/F ratios. The inclusion, exclusion and mechanical ventilation settings in the validated study were like the derivation group. Moreover, ABG measurements and S_P_O_2_ data were collected at similar time points using methods like the derivation data set.

### Statistical analysis

#### Sample size

The sample sizes were determined using data obtained from a pilot investigation (unpublished): derivation (*n* = 30), validation (*n* = 50). Calculations were performed using G*Power version 3.0.10 (Universität Düsseldorf, Germany; available online at http://www.psycho.uni-duesseldorf.de/abteilungen/aap/gpower3/) [14, 15]. Calculations were performed for the variable P/F ratio, with an effect size of 0.3. With an alpha level of 5%, a confidence level of 95%, a power of 90%, and anticipated attrition of 10%, sample sizes of at least 150 and 300 patients were needed in the derivation and validation groups respectively.

#### Data analysis

Normally distributed continuous variables are expressed as the means ± standard deviation (SD). Normally distributed continuous variables were compared using the t-test. Categorical variables were compared using the Chi-Square (χ^2^) and Fisher’s Exact tests as appropriate. The correlation between S/F, P_A_/F and P/F ratios were analyzed using Pearson correlation analysis. Linear regression modeling was utilized to compare the relationship between S/F, P_A_/F and P/F ratios. ROC curves were plotted to determine the prognostic values of the S/F and P_A_/F threshold values correlating with P/F ≤ 300. For each ROC curve analysis, sensitivity, specificity, positive and negative predictive values (PPV and NPV), positive and negative likelihood ratios (LR+ and LR-), and probabilities for having a good surrogate when test is either positive or negative were used to predict the outcome (having good surrogate) in the validation data set.

The Hanley and McNeil method was used to calculate the AUC for each ratio, following which the AUCs were compared using methods that we have previously published [16]. In all analyses, *P* < 0.05 was considered significant. All analyses were performed using IBM® SPSS® v23.0 (IBM Corp., Armonk, NY), STATA 10 (Stata Corp. LLC, College Station, Texas, USA), and GraphPad Prism 5© (Graph Pad Software Inc., La Jolla, CA).

#### Analysis of the derivation data set

Scatter plots of S/F and P_A_/F vs P/F ratios were utilized to determine the linear relationship between the three measurements. A linear regression model was then used to quantify the best regression line. The equation for this regression line was employed to determine the threshold values for S/F and P_A_/F ratio that correlate with P/F ratios consistent with ARDS (P/F ≤ 300). The (S/F)/(P/F) and (P_A_/F)/(P/F) ratios were plotted against F_i_O_2_ and S_P_O_2_ to assess the effect of each on the relationship. ROC curves were plotted to assess the degree of discrimination between S/F and P_A_/F with P/F ratios and to slightly adjust the S/F and P_A_/F ratio threshold values for ARDS to optimize the sensitivity and specificity.

#### Analysis of the validation data set

Linear regression modeling was utilized to quantify the relationship between S/F, P_A_/F and P/F ratios in the validation data set. ROC curves were plotted to determine the sensitivity and specificity of the threshold values derived from the derivation data set for ARDS, with the AUC calculated to assess the degree of discrimination between the ratios.

## Results

**Fig. 1:**
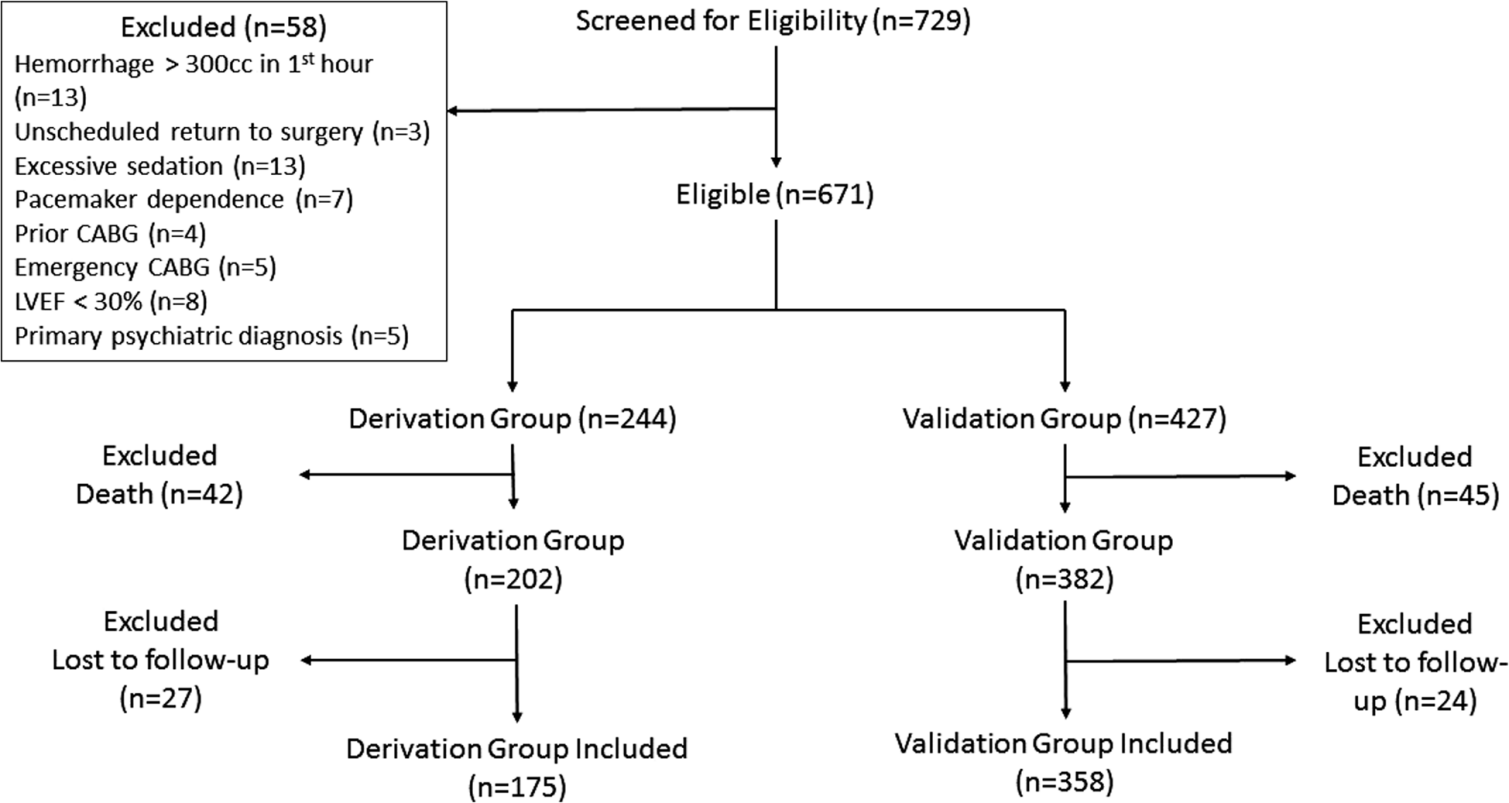
Patient flow diagram

A total of 729 eligible patients were screened for the study. See Figure 1 for the patient flow diagram. Fifty-eight did not meet inclusion criteria. Six-hundred Seventy-one patients consented to participate in the derivation (*n* = 244) and validation (*n* = 427) cohorts. Eighty-seven subjects died (derivation 42, validation 45), 51 were lost to follow-up (derivation 27, validation 24), and 533 were analyzed (derivation 175, validation 358). There were 18 (derivation 7, validation 11) instances where the S_P_O_2_ and ABG could not be sampled at the same time, in which case the nearest S_P_O_2_ was used (all within 30 min). Patient demographics, clinical features, and physiologic respiratory variables are summarized in Table 1. A total of 20 (28.2%) patients were ventilated during cardiopulmonary bypass. The mean pre-op LVEF was 47.75 ± 7.87% (P/F = 48.96 ± 8.60, S/F = 46.04 ± 7.80, P_A_/F = 48.26 ± 7.17; *p* = 0.414). Subjects were transfused a mean of 2.06 ± 0.79 units of packed red cells (P/F mean = 1.92 ± 0.83, S/F *n* = 2.29 ± 0.75, P_A_/F *n* = 1.96 ± 0.77; *p* = 0.20), of which 15 (21.1%) were allogeneic (P/F = 5 (20.8%), S/F = 5 (20.8%), P_A_/F = 5 (21.7%); *p* = 0.996). Six (8.5%) received inotropes (P/F = 3 (12.5%), S/F = 2 (8.3%), P_A_/F = 1 (4.3%); *p* = 0.604). Mean duration of post-operative intra-aortic balloon pump support was 67.38 ± 21.45 h (P/F 67.29 ± 16.68; S/F 70.00 ± 22.99; P_A_/F 64.74 ± 24.61; *p* = 0.71).

**Table 1:** Demographic and clinical data

Variables	Total	Derivation group	Validation group	*P*-value
Age Mean ± SD	70.17 ± 12.17	69.21 ± 12.18	70.64 ± 12.16	0.20
Sex, Female (%)	270 (50.7)	90 (51.4)	180 (50.3)	0.80
DoI, hours Mean ± SD	49.14 ± 27.63	47.12 ± 29.07	50.12 ± 26.88	0.24
P_A_O_2_ Mean ± SD	228.54 ± 24.30	225.83 ± 23.17	229.86 ± 24.75	0.07
P_a_O_2_ Mean ± SD	94.15 ± 3.30	93.21 ± 3.89	94.62 ± 2.87	0.20
S_P_O_2_ Mean ± SD	94.13 ± 1.69	94.05 ± 1.60	94.17 ± 1.75	0.46
pH Mean ± SD	7.41 ± 0.06	7.40 ± 0.06	7.42 ± 0.06	0.07
P_a_CO_2_ Mean ± SD	45.41 ± 3.77	44.71 ± 4.36	45.75 ± 3.39	0.09
F_i_O_2_ Mean ± SD	27.61 ± 2.85	27.6 ± 3.12	27.61 ± 2.72	0.96
Hgb Mean ± SD	11.19 ± 1.52	10.86 ± 1.63	11.35 ± 1.43	< 0.001
P/F Mean ± SD	344.76 ± 38.28	342.09 ± 41.79	346.07 ± 36.42	0.26
P_A_/F Mean ± SD	837.19 ± 126.70	828.94 ± 127.32	841.23 ± 126.37	0.29
S/F Mean ± SD	344.39 ± 34.16	344.78 ± 36.22	384.00 ± 33.21	0.856
OI Mean ± SD	4258.47 ± 809.76	4281.55 ± 765.33	4247.19 ± 831.42	0.65
OSI Mean ± SD	4254.35 ± 796.11	4230.81 ± 703.39	4265.86 ± 838.41	0.63
Survival, alive n (%)	446 (83.7)	150 (85.7)	296 (82.7)	0.37

DoI means duration of intubation; P_A_O_2_ means partial pressure of O_2_ in alveoli; P_a_O_2_means partial pressure of O_2_ in arterial blood; S_p_O_2_ means peripheral capillary oxygen saturation; P_a_CO_2_ means partial pressure of CO_2_ in arterial blood; F_i_O_2_ means fraction of inspired oxygen; Hgb means hemoglobin; S/F means S_P_O_2_/FiO_2_ ratio; P/F means P_a_O_2_/FiO_2_ratio; P_A_/F means P_A_O_2_/FiO_2_ ratio; OI means Oxygen Index; OSI means Oxygen Saturation Index

Our data met the criteria only for the PF ratio for mild ARDS (PF 200–300). The mean time between charted P_a_O_2_ and S_P_O_2_ pairs was 50.27 ± 11.65 s (Derivation 50.21 ± 12.18; Validation 50.30 ± 11.40).

**Fig. 2:**
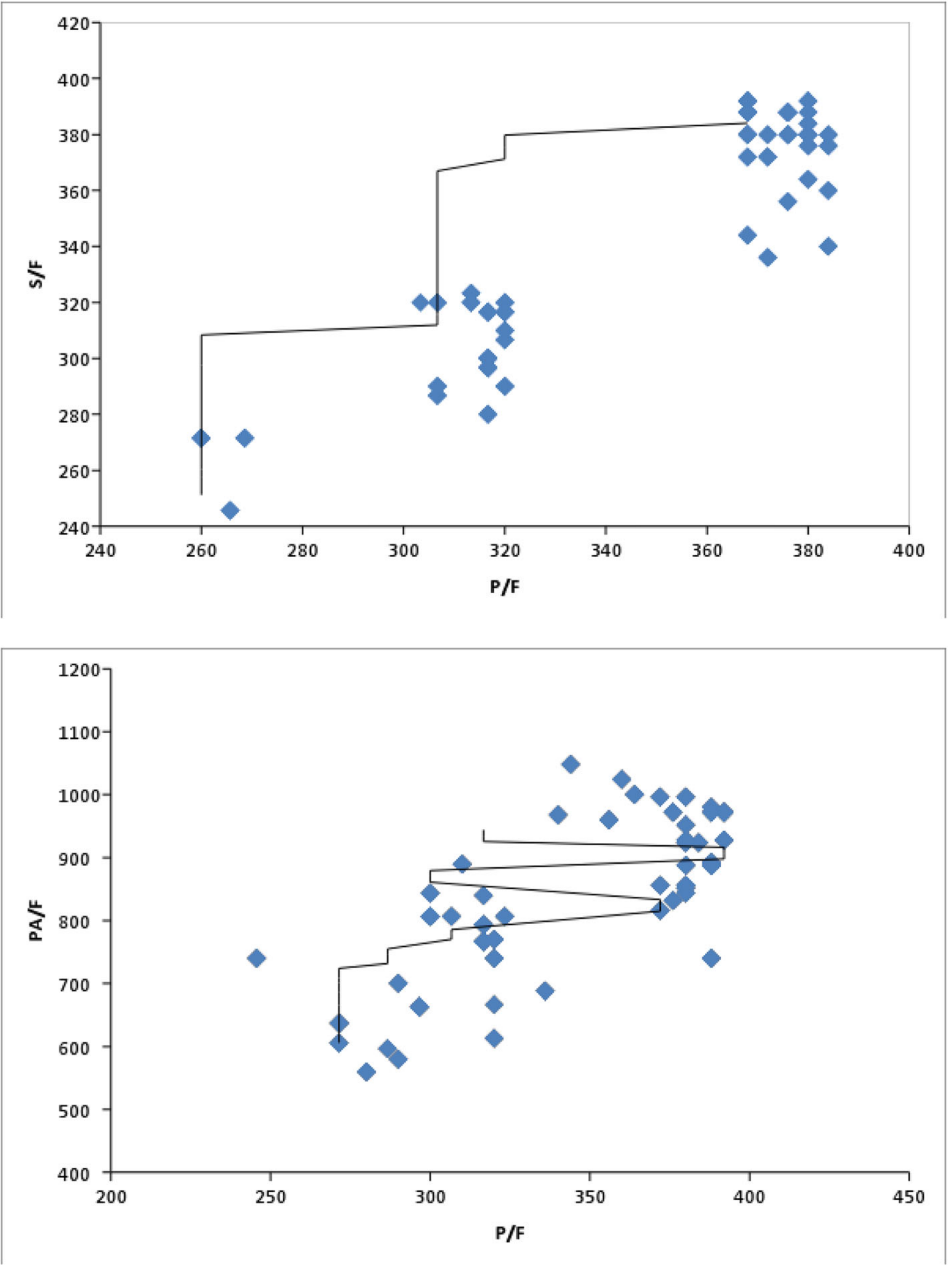
Scatter plots for S/F vs. P/F ratios (top) and P_A_/F vs. P/F ratios (bottom) for the derivation data set. The S/F vs. P/F line of best fit equals (S/F = 71.149 + 0.8PF); (*P* < 0.0001; *r* = 0.926); whereas the P_A_/F vs. P/F line of best fit equals (P_A_/F = 38.098 + 2.312PF); (*P* < 0.0001; *r* = 0.759)

**Fig. 3:**
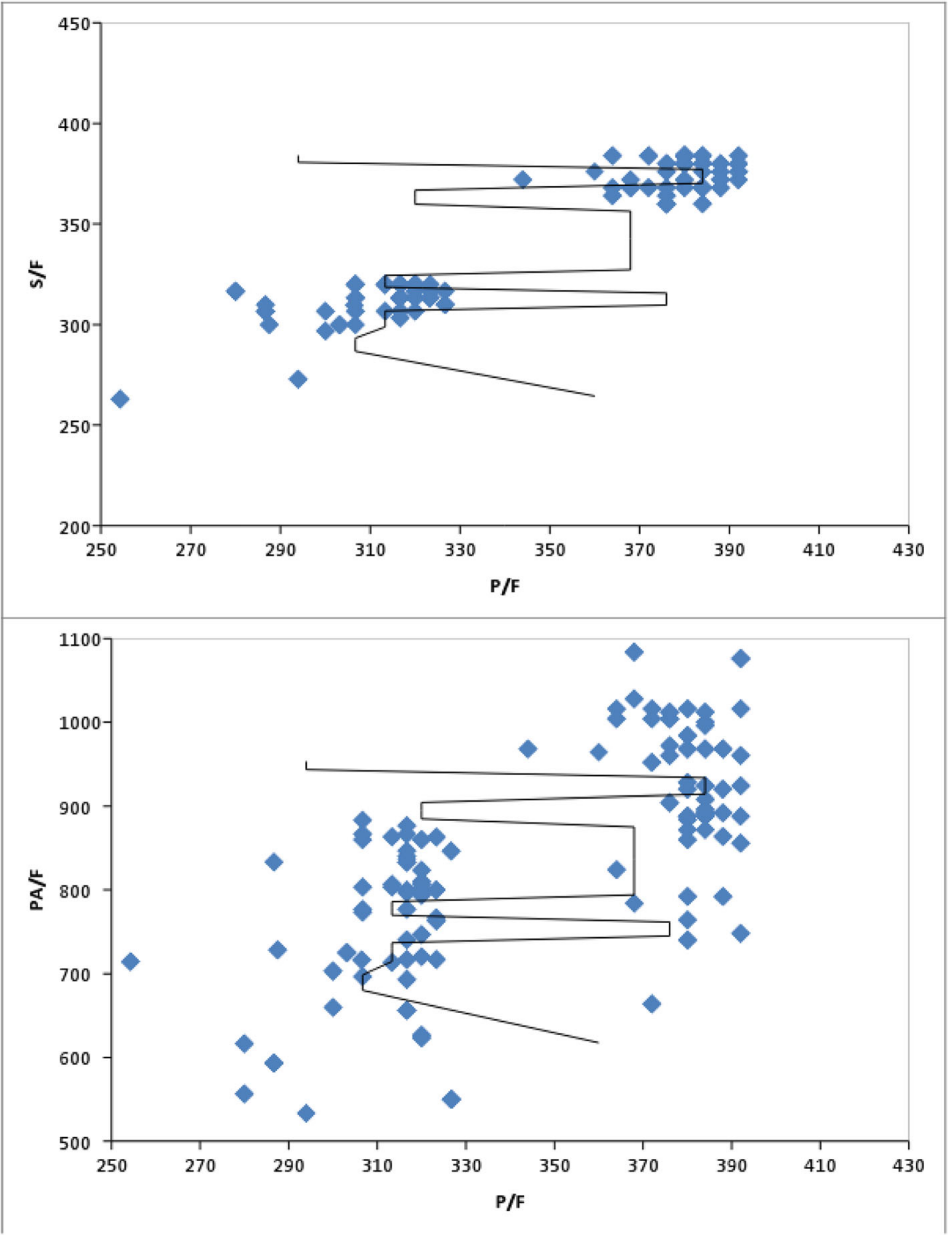
Scatter plots for S/F vs. P/F ratios (top) and P_A_/F vs. P/F ratios (bottom) for the validation data set. The S/F vs. P/F line of best fit equals (S/F = 43.309 + 0.869PF); (R^2^ 0.909; F 3565.427; < 0.0001); (< 0.0001; *r* = 0.954); whereas the P_A_/F vs. P/F line of best fit equals (PAFR = − 1.537 + 2.435PFR); (R^2^ 0.493; F 345.762; < 0.0001); (< 0.0001; *r* = 0.702)

In the derivation cohort, the S/F and P_A_/F ratios could be predicted well from P/F, described by the linear regression models S/F = 71.149 + 0.8PF and P_A_/F = 38.098 + 2.312PF, respectively. According to the linear regression equation, a P/F ratio of 300 equaled an S/F ratio of 311 (R^2^ 0.857, F 1035.742, < 0.0001) and a P_A_/F ratio of 732 (R^2^ 0.576, F 234.887, < 0.0001). The scatter plots for the (S/F vs P/F) and (P_A_/F vs. P/F) ratios for the derivation and validation data sets is shown in Figs. 2 and 3 respectively.

The S/F threshold of 311 had 90% sensitivity, 80% specificity, LR+ 4.50, LR- 0.13, PPV 98, and NPV 42.1 for the diagnosis of mild ARDS. The P_A_/F threshold of 732 had 86% sensitivity, 90% specificity, LR+ 8.45, LR- 0.16, PPV 98.9, and NPV 36 for the diagnosis of mild ARDS. S/F had excellent discrimination ability for mild ARDS (AUC ± SE = 0.92 ± 0.017; 95% CI 0.889 to 0.947) as did P_A_/F for mild ARDS (AUC ± SE = 0.915 ± 0.018; 95% CI 0.881 to0.942).

## Discussion

ARDS manifests as acute refractory hypoxemia, bilateral pulmonary infiltrates and non-cardiogenic pulmonary edema, and has a very high mortality [23]. The Berlin Criteria (2012) [13, 24] defines ARDS by 4 main characteristics: (1) *Timing* within 1 week of known clinical insult or new or worsening symptoms; (2) *Chest imaging* with bilateral opacities not fully explained by effusions, lobar/lung collapse, or nodules; (3) *Origin of edema* not fully explained by cardiac failure or fluid overload; and *Oxygen* impairment defined as mild (200 mmHg < P/F ≤ 300 mmHg, with PEEP or CPAP ≥5 cmH_2_O), moderate (100 mmHg < P/F ≤ 200 mmHg, with PEEP ≥5 cmH_2_O), or severe (P/F ≤ 100 mmHg, with PEEP ≥5 cmH_2_O).

It is known, however, that ARDS criteria may be met in post-op CABG patients in the absence of true ARDS. Thus, a need exists to improve diagnostic specificity [25]. Owing to concerns about anemia, excessive phlebotomy, and a movement to minimally invasive approaches, we investigated whether non-invasive indices of oxygenation (S/F, P_A_/F) performed equal to or better than P/F in identifying post-operative ARDS in patients post-CABG.

Despite wide recognition of the high morbidity and mortality associated with ARDS, few interventions have been observed to decrease either. One reason for this may be late or delayed diagnosis, rendering the S/F ratio a rapid, convenient, and useful diagnostic tool. A study by of 1742 matched measurements of S_p_O_2_ and P_a_O_2_ in adult patients undergoing general anesthesia sought to identify a correlation between the S/F and P/F ratios by incorporating the S/F into the respiratory component of the SOFA score and found that both predicted similar outcomes [26]. Results were less convincing in the pediatric population, where only a weak correlation between S/F and P/F was noted in children with acute lung injury (age 1 month to 18 years) [27]. In an analysis of patients from the ARDS Network, S/F ratios of 235 and 315 correlated with PF ratios of 200 and 300 respectively [20]. Our findings were in keeping with prior reports that S/F ratios correlate with P/F ratios [18, 28–30], supporting our finding that an S/F ratio of 311 correlates with a P/F of 300 (Sensitivity 90%, Specificity 80%).

Advantages of using the S/F (aka S_p_O_2_/F_i_O_2_) ratio in the diagnosis of ARDS are many. It may allow for real-time monitoring, is dynamic allowing for rapid identification of oxygenation changes, and may allow for earlier diagnosis. Additionally, it allows for a decrease in phlebotomy, blood loss, fewer skin breaks, fewer vascular punctures, and less associated pain. Furthermore, it is affordable, time saving, and already standard practice in most ICUs [31, 32].

Despite obvious advantages and utility of S/F, its use may not be appropriate for all situations. For example, some factors that limit the accuracy of pulse oximetry include methemoglobinemia, oximeter location, cardiogenic shock and temperature [30]. Other factors to consider include patient position andcognitive status (eg. delirium, agitation), which may alter measurements due to movement. Therefore, to decrease the risk of error and resultant misdiagnosis, a steady wave form as described in our study should be used.

## Conclusion

In conclusion, cardiac surgery with cardiopulmonary bypass elicits a systemic inflammatory response that increases the risk for ARDS. P_a_O_2_ and S_a_O_2_ correlated in the diagnosis of ARDS, with a P/F of 300 correlating to an S/F of 311 (Sensitivity 90%, Specificity 80%). The S/F ratio may allow for early real-time rapid identification of ARDS, while decreasing the cost, phlebotomy, blood loss, pain, skin breaks, and vascular punctures associated with ABG measurements.

## Abbreviations

ABG: Arterial blood gas
ARDS: Acute respiratory distress syndrome
AUC: Area under the curve
CABG: Coronary artery bypasses graft
CAD: Coronary artery disease
CVD: Cardiovascular disease
F_i_O_2_: Fraction of inspired oxygen
LVEF: Left ventricular ejection fraction
P/F: P_a_O_2_/FiO_2_
P_A_/F: P_A_O_2_/FiO_2_
P_A_O_2_: Partial pressure of O_2_ in alveoli
P_a_O_2_: Partial pressure of O_2_ in arterial blood
PBW: Predicted body weight
ROC: Receiver operating characteristic
S/F: S_P_O_2_/FiO_2_
SIRS: Systemic inflammatory response syndrome
S_p_O_2_: Peripheral capillary oxygen saturation
V_T_: Tidal volume
